# Translocation of deadwood in ecological compensation: A novel way to compensate for habitat loss

**DOI:** 10.1007/s13280-023-01934-0

**Published:** 2023-10-11

**Authors:** Olov Tranberg, Anne-Maarit Hekkala, Ola Lindroos, Therese Löfroth, Mari Jönsson, Jörgen Sjögren, Joakim Hjältén

**Affiliations:** 1 Department of Wildlife, Fish, and Environmental Studies, Swedish University of Agricultural Sciences, 901 83 Umeå, Sweden; 2 Department of Forest Biomaterials and Technology, Swedish University of Agricultural Sciences, 901 83 Umeå, Sweden; 3 SLU Swedish Species Information Centre, Swedish University of Agricultural Sciences, Uppsala, Sweden

**Keywords:** Biodiversity offset, Boreal forest, Conservation, Cost-efficiency, Deadwood, Restoration

## Abstract

Restoration of degraded habitat is frequently used in ecological compensation. However, ecological restoration suffers from innate problems of long delivery times of features shown to be good proxies for biodiversity, e.g., large dead trees. We tested a possible way to circumvent this problem; the translocation of hard-to-come deadwood substrates from an impact area to a compensation area. Following translocation, deadwood density in the compensation area was locally equivalent to the impact area, around 20 m^3^ ha^−1^, a threshold for supporting high biodiversity of rare and red-listed species. However, deadwood composition differed between the impact and compensation area, showing a need to include more deadwood types, e.g., late decomposition deadwood, in the translocation scheme. To guide future compensation efforts, the cost for translocation at different spatial scales was calculated. We conclude that translocation of deadwood could provide a cost-efficient new tool for ecological compensation/restoration but that the method needs refinement.

## Introduction

Exploitation of forest ecosystem has led to changes in ecosystem structures and processes, and to biodiversity loss (FAO 2010; Ceballos et al. 2015). To simultaneously conserve biodiversity and continue economic development is a major challenge for human society (Lubchenco 1998). While sustainable development depends on functional ecosystems in numerous ways, economic growth and biodiversity conservation are often perceived to be incompatible.

Within this context there is an increasing pressure on corporations by consumers and stakeholders to be environmentally conscious and more focus is directed towards alternative approaches in adapting to this demand (Sjåfjell 2012; Verrier et al. 2014). One such approach is the relatively recent concept of ecological compensation (biodiversity offsetting) which is based on the principle that those who damage or destroy natural values are to compensate for the loss by generating or protecting natural values at a different/substitute location (polluters-pay-principle) (OECD 1992; Bull et al. 2013). Thus, ecological compensation, at least in theory, provides an approach to allow economically important human development while ensuring that ‘no-net-loss’, or even ‘net positive gain’, in biodiversity is achieved (Bull et al. 2013; Gardner et al. 2013). Although legislation mandating ecological compensation, as a final measure in mitigating negative impact by exploitations, exist in many countries, principles and methods for biodiversity offsetting are still under development (Koh et al. 2017; Blicharska et al. 2022). Methods for ecological compensation can involve protection of areas that are otherwise at risk of exploitation, ecological restoration or other positive management interventions and, in some circumstances, the recreation of habitat that has been lost.

Restoration of degraded habitat is often used in ecological compensation and our knowledge of the effects of different restoration methods on biodiversity has improved in recent years (Berglund et al. 2011; Halme et al. 2013; Hekkala et al. 2014; Hjältén et al. 2017, 2023). However, restoration often suffers from the innate problem that, even if in situ restoration provides substrates or habitat for species that we want to favor, those species may not be able to disperse to restored habitats or areas (Kouki et al. 2012; Bell et al. 2015), often referred to as “field-of-dreams” dilemma (Palmer et al. 1997; Hilderbrand et al. 2005). Furthermore, the delivery time on certain types of habitats is very long, several hundreds of years for, e.g., live and dead large-diameter trees and advanced decay classes of deadwood. Thus, the loss of these kinds of habitats are difficult to compensate for in ecological compensation. One potential approach to circumvent problems with dispersal and long delivery time is the translocation of some of these unique substrates together with associated species.

Such translocation of deadwood can potentially play an important role in ecological compensation, as it constitutes a key habitat for biodiversity in the boreal forest. Decrease in deadwood availability and diversity due to forestry and other types of land use is the main explanation for loss of biodiversity on saproxylic species (Siitonen 2001; Stokland et al. 2012; Löfroth et al. 2023). Species richness and ecological communities of deadwood dependent species (insects, wood fungi, bryophytes and lichens, and indirectly also top predators such as woodpeckers) is determined by amount (abundance and volume) and diversity of deadwood in terms of tree species, trunk size, posture, mortality factor (e.g., wind, fire) and stage of decomposition (Siitonen 2001; Similä et al. 2003; Junninen and Komonen 2011; Seibold et al. 2016; Hägglund and Hjältén 2018; Kärvemo et al. 2021). In general, high amounts of deadwood have shown to be good a proxy for biodiversity with 20 m^3^ ha^−1^ serving as a threshold for maintaining high species richness of rare and red-listed saproxylic species, e.g. fungi, in boreal forests (Penttilä et al. 2004; Hekkala et al. 2023). In addition, the volume of deadwood has been identified as one of the EU-level indicators used to quantify the state of forests’ biological diversity (Bozzano and Oggioni 2020). Maintaining high volumes and diversity of deadwood is therefore crucial for saproxylic biodiversity.

Still, translocation of deadwood has rarely been conducted at large scale and the method has to the best of our knowledge never been scientifically evaluated. In theory, translocation of deadwood offers a rapid establishment of high-quality habitat and assisted migration of various deadwood dependent species, communities that might take long time to colonize through natural processes (Morris et al. 2006; Fenton and Bergeron 2008; Toivanen and Kotiaho 2010). In contrast creating deadwood in situ, although a slow process, result in a more gradual establishment of habitats and communities (Toivanen and Kotiaho 2007; Djupström et al. 2012). In situ creation of deadwood also generally requires less resources compared to translocation. Therefore, there is a need to assess if, in practice, the translocation can result in similar densities and compositions of deadwood between the area they were translocated from and the area they were moved to. We are also lacking knowledge of what constitutes feasible scales for translocation of deadwood, in terms of the costs incurred and deadwood amounts needed when translocating to plots, forest stands and landscapes (Lindroos et al. 2021). This information is needed even for the assessment of the value of deadwood translocation compensation for related associated biodiversity.

There is also a need to assess the cost of compensation measures as this will impact if they will be implemented or not. When costs for ecological compensation projects have been investigated, it has often been in terms of the total costs for compensation projects carried out. However, there are also some research focusing on making it possible to compare alternatives to find and develop cost-efficient practices (e.g., Cuperus et al. 2001; Lindroos et al. 2021). However, the costs of deadwood translocation to different spatial scales have never been investigated. Even when the ecological compensation constitutes a minor part of large-scale projects, such as the construction of roads and establishment of mines, cost-efficiency is, nevertheless, instrumental for increasing both the use of ecological compensation and increasing the benefits from a given economic input.

A large-scale experiment, using a before-after-control-impact approach, was initiated in 2016 to assess the effects of translocating deadwood from a high conservation value forest (impact area, subjected to exploitation due to expansion of the Aitik mine) to a compensation area with lower conservation value on important habitat characteristics and the costs of translocation. This experiment is exceptional in its magnitude and standard, as 637 deadwood substrates, including various qualities of deadwood such as very old and uncommon types and different tree species, were relocated to a nearby compensation area.

The main objective of this study was to assess if translocation of dead trees to a lower quality forest landscape assigned as compensation area can be used to re-create the deadwood amount, diversity and composition found in the impact area and thus above suggested thresholds for maintaining species richness of rare and red-listed saproxylic species at different scales. Furthermore, to guide future compensation efforts we calculated costs for translocation at different spatial scales.

## Materials and methods

### Study area and design

The study area belong to the north boreal vegetation zone (Ahti et al. 1968) and all sites included in the study have previously been under management, predominantly subjected to selective felling, but have not been managed during the latest decades. The forests are conifer dominated bilberry type or mixed forests (conifers + broadleaves) dominated by Norway spruce [*Picea abies* (L*.*) Karst.] and Scots pine (*Pinus sylvestris* L.) with scattered occurrence of mainly Downy birch (*Betula pubescens* Ehrh.) and Goat willow (*Salix caprea* L.).

**Fig. 1:**
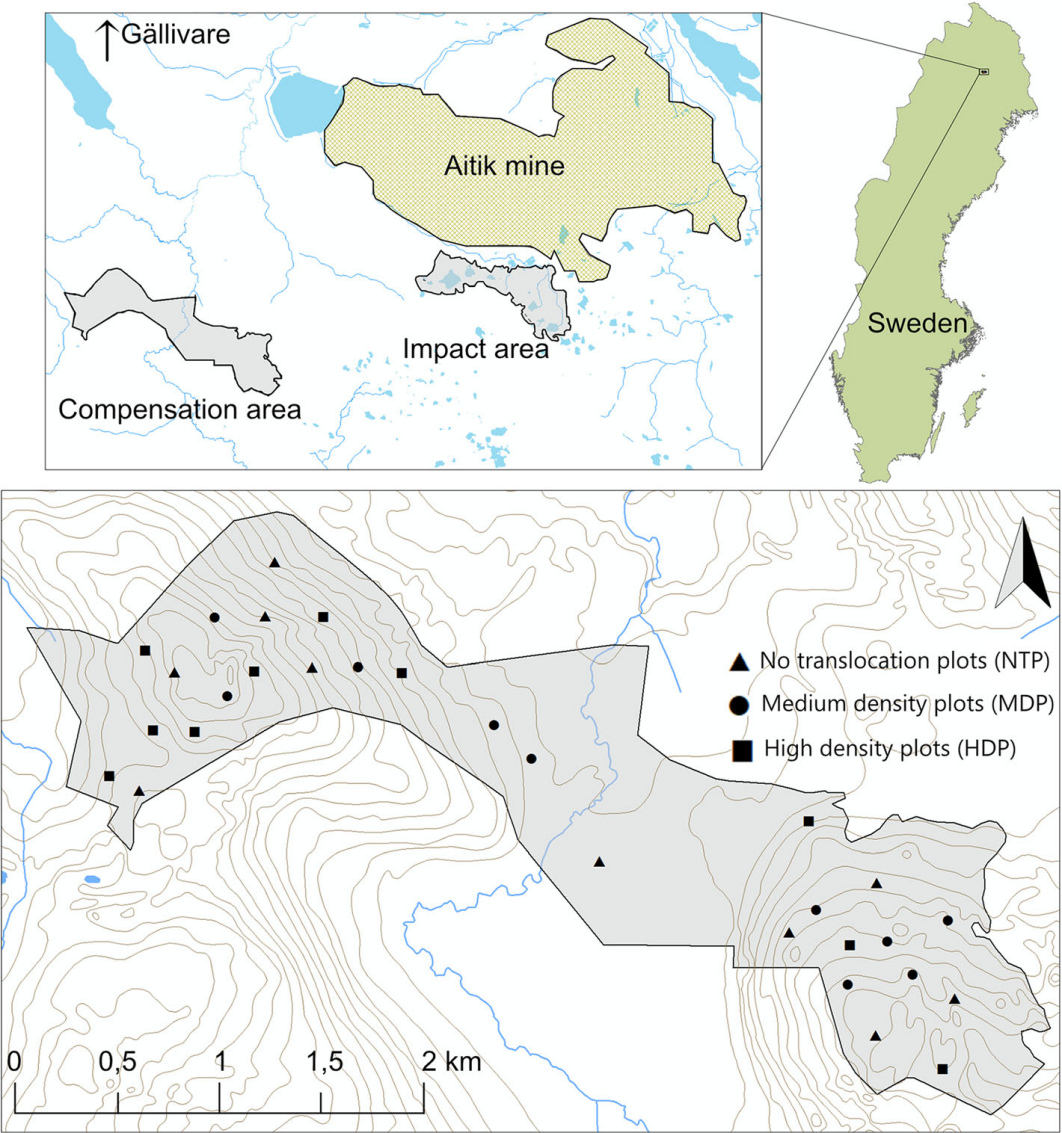
Map of Sweden showing the two sites in the study: impact and compensation area, denoted in grey. The impact area includes forest of high or very high conservation values. Symbols in lower figure indicate plot type in the compensation area

The study includes two sites; an impact area and a compensation area. The impact area encompassed 376 ha out of which 167 ha consisted of forests of high or very high conservation values (by definition of assessment by Swedish Standards Institute (2014), including high volumes of deadwood (mean 21.1 m^3^ ha^−1^) and occurrence of 16 red-listed species of wood fungi and lichens (Forsgren et al. 2016). Remaining 209 ha in the impact area consisted of forest of lower conservation values (144 ha) and non-productive forest, mires or open water. The compensation area encompassed 397 ha, out of which 192 ha had high conservation values (no forest of very high conservation value), with moderate volumes of deadwood (mean 9.3 m^3^ ha^−1^) and occurrence of 11 red-listed species of wood fungi and lichens. Remaining 205 ha of the compensation area consisted of forests of low conservation value (113 ha) and non-productive forest, mires or open water (Forsgren et al. 2016) (Fig. 1).

In 2016, prior to translocation, 10 experimental plots were established in the impact area, each with a radius of 25 m, distributed randomly across productive forestland of higher conservation value. In the compensation area, 30 equally sized plots were created and randomly assigned to one of three groups: no translocation (NTP, *n* = 10), medium density translocation (MDP, *n* = 10), and high density translocation (HDP, *n* = 10). All plots were situated at least 150 m apart from one another. This design was implemented to evaluate the response of wood-living organisms to translocation of different densities of deadwood. Translocation was performed in autumn 2017 and post-translocation measurement was performed in spring 2018.

### Translocation method

**Fig. 2:**
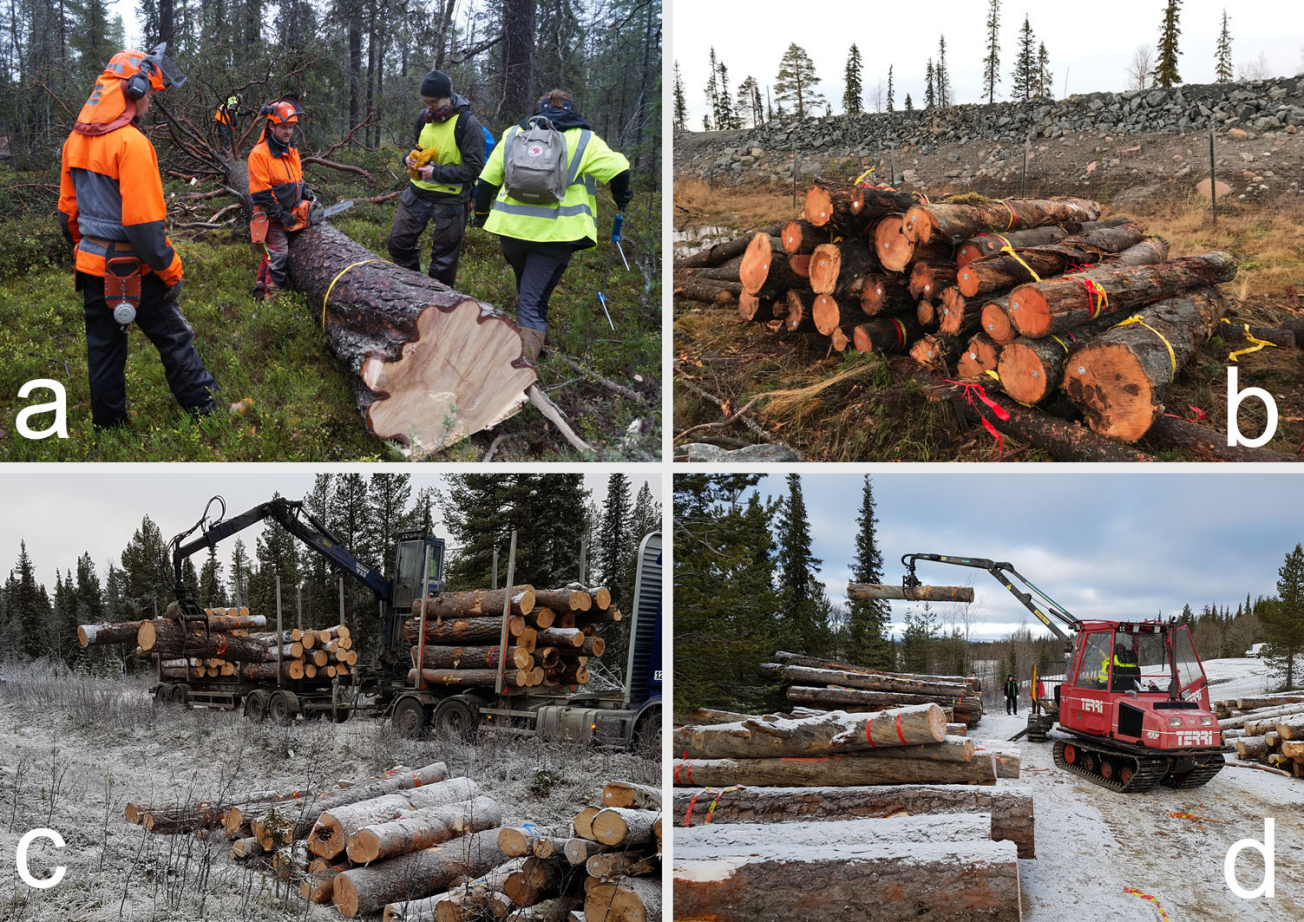
Steps in the translocation process; **a** selection of substrates, **b** marking and storing of substrates, **c** transport to the compensation area, and **d** forwarding to compensation/experimental plots. *Photo* Maria Nordlund (**a**–**b**) and Joakim Hjältén (**c**–**d**)

The translocation of deadwood followed a seven-step scheme: (1) identification of suitable compensation area, (2) identification of suitable deadwood objects (large-end diameter ≥ 25 cm) logs and living trees of high conservation value to be translocated, (3) cutting and bucking of selected logs and trees, (4) ID marking of the selected substrates, (5) extraction of the substrates from impact forest to road, (6) road transport from impact area to compensation area and (7) insertion from road into compensation forest (Fig. 2). More details about the translocation work can be found in Lindroos et al. (2021).

Eight types of suitable deadwood substrates, of Norway spruce and Scots pine, were identified and used for selection (Table 1) from the entire impact area. Only deadwood substrates that were feasible to move without breaking were selected, which includes only downed deadwood in decomposition classes 1–3 and standing dead trees in decomposition classes 3–7 (classes according to Thomas and Parker 1979). The upward-facing part of the selected downed deadwood was marked with color to be able to reset the substrate in the same posture after translocation. A total of 637 deadwood substrates were selected (mean volume 0.292 m^3^), cut and bucked to a length of 3–5 m. The goal was to translocate 80 substrates from each of the eight substrate groups, but due to insufficient numbers of substrates of pine logs, additional substrates were supplemented from standing dead trees and living trees of pine (Table 1). All translocated substrates ended up as downed deadwood in the compensation area.

**Table 1:** Translocated substrates from the impact area were categorized into eight groups based on two tree species, three posture types, decomposition classes, and length. The table presents the distribution of substrates in the impact area, which were translocated to the compensation area and divided into 30 experimental plots; No Translocation Plot (NTP), Medium Density Plots (MDP), and High Density Plots (HDP). A total of 637 substrates were identified, cut, permanently marked, and translocated. DC-class stands for Decomposition Class according to Thomas and Parker (1979)

Substrate type	Tree species	Posture, before translocation	DC-class	Allowed substrate length (m)	Number of received translocated substrates/plot in compensation area			Total (*n* = 30 plots)
					NTP (*n* = 10)	MDP (*n* = 10)	HDP (*n* = 10)	
Translocated substrates	Pine	Downed	1	4 ± 1	–	1 ± 1	1 ± 1	18
	Pine	Downed	2–3	3	–	1 ± 1	4 ± 2	65
	Pine	Standing dead	3–7	4 ± 1	–	3 ± 1	5 ± 2	97
	Pine	Living	1	4 ± 1	–	3 ± 1	8 ± 3	133
	Spruce	Downed	1	4 ± 1	–	2	6 ± 2	80
	Spruce	Downed	2–3	3	–	2 ± 1	6 ± 1	79
	Spruce	Standing dead	3–7	4 ± 1	–	2 ± 1	6 ± 1	81
	Spruce	Living	1	4 ± 1	–	2 ± 1	6 ± 1	84
Total					0	16 ± 1	48 ± 1	637
Trees cut on site					2	2	2	60

Within the compensation area, each plot assigned to the medium density translocation group (MDP) received 16 translocated deadwood substrates, approximately two of each substrate type (Table 1), while each plot assigned to HDP received 48 translocated deadwood substrates, approximately 6 of each substrate type. No deadwood substrates were translocated to the NTPs. Additionally, in all compensation area plots, one living pine and one living spruce tree were cut and left unbucked to allow for evaluating future colonization of saproxylic species on the deadwood.

### Field measurements of deadwood

In 2016, prior to exploitation and translocation, measurements of deadwood were conducted in the impact area and the compensation area. Deadwood characteristics were measured (Table 2) from the 25 m radius plot for all naturally occurring dead trees with minimum large-end diameter ≥ 5 cm, including base and top diameter, tree species and trunk length. Posture of each tree was determined into two classes, standing dead trees or downed logs. Trees originated from outside the plots were not measured. Height and DBH of standing dead trees and snags were measured. Decomposition class (DC) for coniferous deadwood was determined by the classification system derived from Thomas and Parker (1979), including classes DC1–5 for downed and DC1–7 for standing deadwood. For broadleaves decomposition class was determined by the classification system from Gibb et al. (2005) into deadwood softness (“hard” or “soft”). The deadwood inventories were repeated in 2018 after translocation, thus including measures of both naturally occurring and translocated deadwood.

**Table 2:** PERMANOVA analyses (ADONIS) results for the effects and significance of differences in the deadwood composition in the five studied translocation groups/treatments. Response categories represent the different plot types in comparison

Response category 1	Response category 2	*R*^2^	*p*-value
Impact area			
	Compensation area before	0.161	< 0.001
	No translocation plots	0.127	0.003
	Medium density plots	0.407	< 0.001
	High density plots	0.532	< 0.001
No translocation plots			
	Medium density plots	0.436	< 0.001
	High density plots	0.567	< 0.001
Medium density plots			
	High density plots	0.573	< 0.001

### Data processing and analysis

Deadwood volume for logs were calculated using the formula for a truncated cone (*V*_t_) where *L* is the length, *r*_max_ the maximum radius and *r*_min_ the minimum radius.

$${V}_{\mathrm{t}}=\left(L* \frac{\pi }{3}\right)*\left({{r}_{\mathrm{max}}}^{2}+{r}_{\mathrm{max}}*{{r}_{\mathrm{min}}}+{{r}_{\mathrm{min}}}^{2}\right).$$

The deadwood volume for whole standing dead trees (snags) was calculated using functions from Näslund (1940) with specific functions for pine, spruce and birch. Deadwood volume for high stumps or broken trees was calculated using the formula for a cylinder (*V*_c_) where h marks height of the high stump and *r* the DBH divided in two.

$${V}_{\mathrm{c}}=\pi {r}^{2}*h.$$

Using the conversion method by Thomas and Parker (1979) we converted decomposition class for standing deadwood (DC1–7) into corresponding class of downed deadwood (DC1–5) to make one consistent decomposition class system. For the same reason, the decay class for birch and willow was converted from softness of deadwood into corresponding decay classes (DC3–4) derived from Thomas and Parker (1979).

Using linear regression, we tested for differences in deadwood volumes between the plots in the impact area and the different translocation plots, followed by Tukey pairwise post hoc multiple comparisons of means on a 95% family wise confidence level. We calculated volume of deadwood on plot (radius of 25 m), stand (1 ha) and landscape scale (up to 500 ha) to assess the cost and effort needed for compensation at different spatial scales. To determine deadwood volume on plot scale we measured both the initial deadwood volume before translocation and the translocated deadwood. To estimate deadwood volume on stand scale, we multiplied the initial volume before translocation for each plot and treatment (NTP, HDP, and MDP) by the area of one hectare, followed by addition of the translocated deadwood volumes to each treatment. To evaluate the effect of deadwood addition on larger scales we calculated how much deadwood would be needed to enrich a landscape up to 500 ha. The needed deadwood enrichment was calculated as the difference between the deadwood volume in the impact area minus the background level in the compensation area before translocation.

In order to compute the costs of alternative intensities and sizes of areas for compensation measures, data from the executed translocation work and derived models reported in Lindroos et al. (2021) were used. The costs were set to fixed values for area identification (33.4 SEK/log), for substrate identification (73.8 SEK/log) and for felling (164.1 SEK/log). For the transport related work, the cost was a function of the hourly cost for the work, the load size and the transport distance. For extraction, the hourly cost was set to 900 SEK, to 750 SEK for road transport and to 900 SEK for insertion. Load size was set to 18 logs in extraction, 105 logs in road transport and 10 logs in insertion. The road transport distance was set to 24 km, whereas the extraction and insertion distances depended on the area of the assumed impact and compensation areas. For simplicity, it was assumed that the impact and compensation areas were of the same sizes, circular and located right next to roads. Extraction and insertion distances were therefore identical, and equivalent to the radius of a circle with the given area.

We examined the diversity of deadwood substrates, including both translocated and natural occurring deadwood, in the impact area compared with the two different types of translocation plots (MPD and HDP). We generated specific deadwood substrate groups using all possible combinations of four selected deadwood variables including *tree species*, *decomposition class*, *diameter class* (10 cm intervals) and *type* (snag or downed), resulting in 240 possible unique deadwood types. The count of unique deadwood types per plot was considered as deadwood diversity on the plot. Diversity patterns were furthermore visualized with NMDS using unique deadwood types as species, followed by PERMANOVA, using the function *adonis* in the R-package Vegan (Oksanen et al. 2020). To examine specific deadwood types with significant higher occurrence (presence/absence) in any of the translocation plots or study areas, we used indicator species analysis from the *indicspecies*-package (De Cáceres et al. 2022). All statistical calculations and analyses were performed with R software (R Core Team 2021).

## Results

### Deadwood volume

**Fig. 3:**
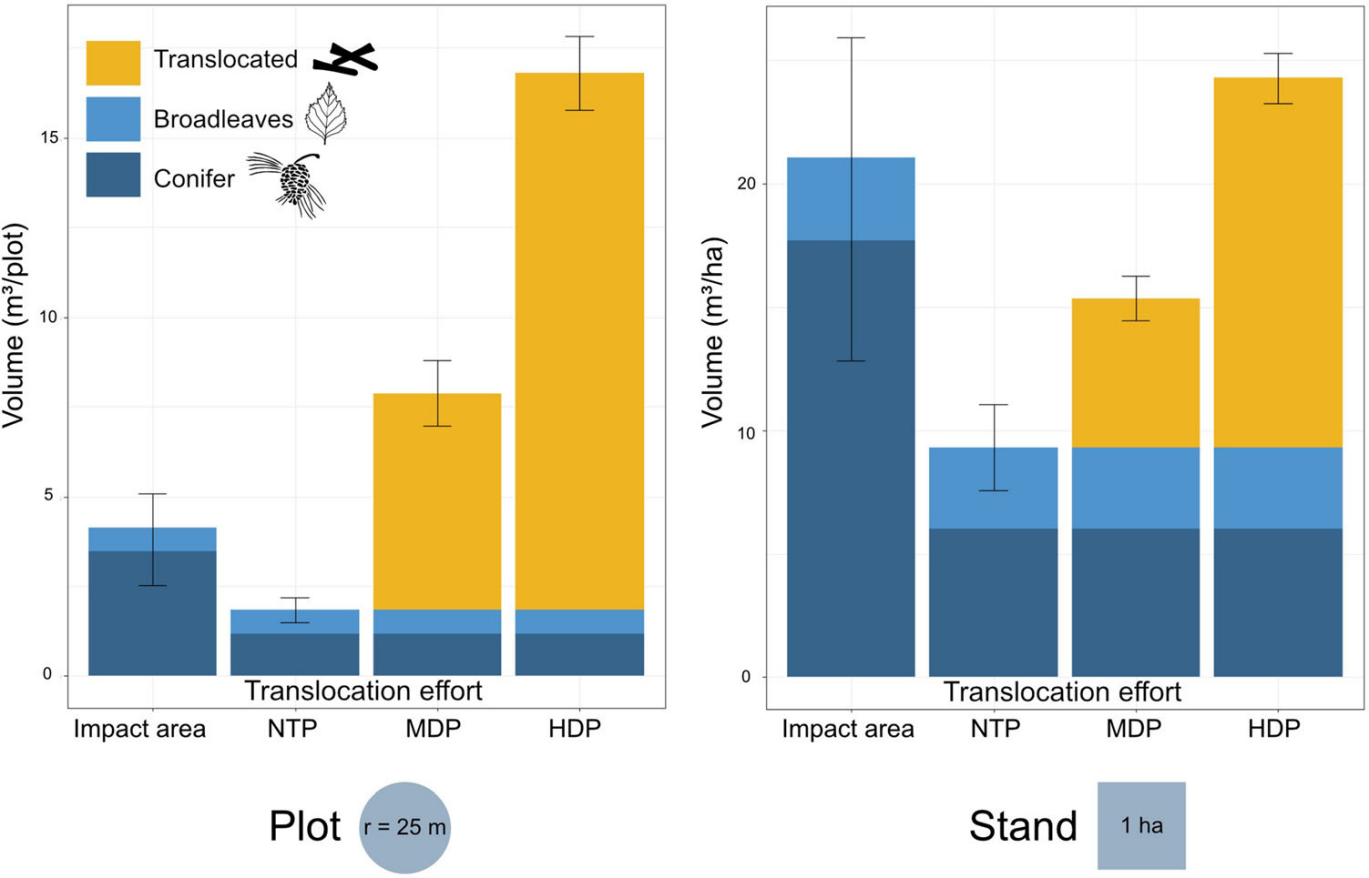
Deadwood volumes in the impact area (far left) and compensation area, the later divided into NTP, no translocation plots (no deadwood addition); MDP, medium density plots (addition of 16 substrates/plot) and HDP, high density plots (addition of 48 substrates/plot). Translocated deadwood (yellow) consist only of conifers

On average, the compensation area plots had half the volume of deadwood prior to translocation when compared to the impact area plots (1.8 and 4.1 m^3^ plot^−1^, respectively). Following translocation, HDP had significantly higher volumes (*p* < 0.001) than the other plot types, while MDP had higher volumes than NTP (*p* < 0.001), as illustrated in Fig. 3. After translocation, both HDPs and MDPs had higher total deadwood volumes than the impact area (*p* < 0.001 for both). Translocated deadwood accounted for 89%, 76%, and 0% of the total volume of deadwood in HDP, MDP, and NTP, respectively.

Neither of the two translocation plot types, MDP and HDP, had significantly different volume compared to the impact area at the stand level (MDP = 15.4 ± 1.8, HDP = 24.3 ± 2.0 and impact = 21.1 ± 4.8 m^3^ ha^−1^, respectively, and *p* = 0.21 for MDP and *p* = 0.12 for HDP, respectively). Translocated deadwood accounted for 61%, 39%, and 0% of the total volume of deadwood in HDP, MDP, and NTP, respectively, as shown in Fig. 3.

On smaller spatial scales, such as plot and stand level, the executed compensations resulted in similar deadwood volumes as those found in the impact area (Fig. 3). However, when accounting for the full impact area, the executed compensations resulted in a considerable deadwood shortage compared to the amount of deadwood in the impact area. In total 637 logs were translocated to the compensation area of 310 ha (excluding mires and open water), which equals a deadwood addition of on average 2.1 logs ha^−1^, or 0.58 m^3^ ha^−1^ to the 9.3 m^3^ ha^−1^ already present in the compensation area. This should be contrasted to 21.1 ± 4.8 m^3^ ha^−1^ of deadwood found in the impact area, which is equal to 72.2 ± 16.4 logs ha^−1^. To fully reach levels similar to those in the entire impact area, an additional 7.0–16.6 m^3^ ha^−1^, equivalent to 23.9–56.8 logs ha^−1^, of deadwood would be needed when taking the entire compensation area in consideration.

**Fig. 4:**
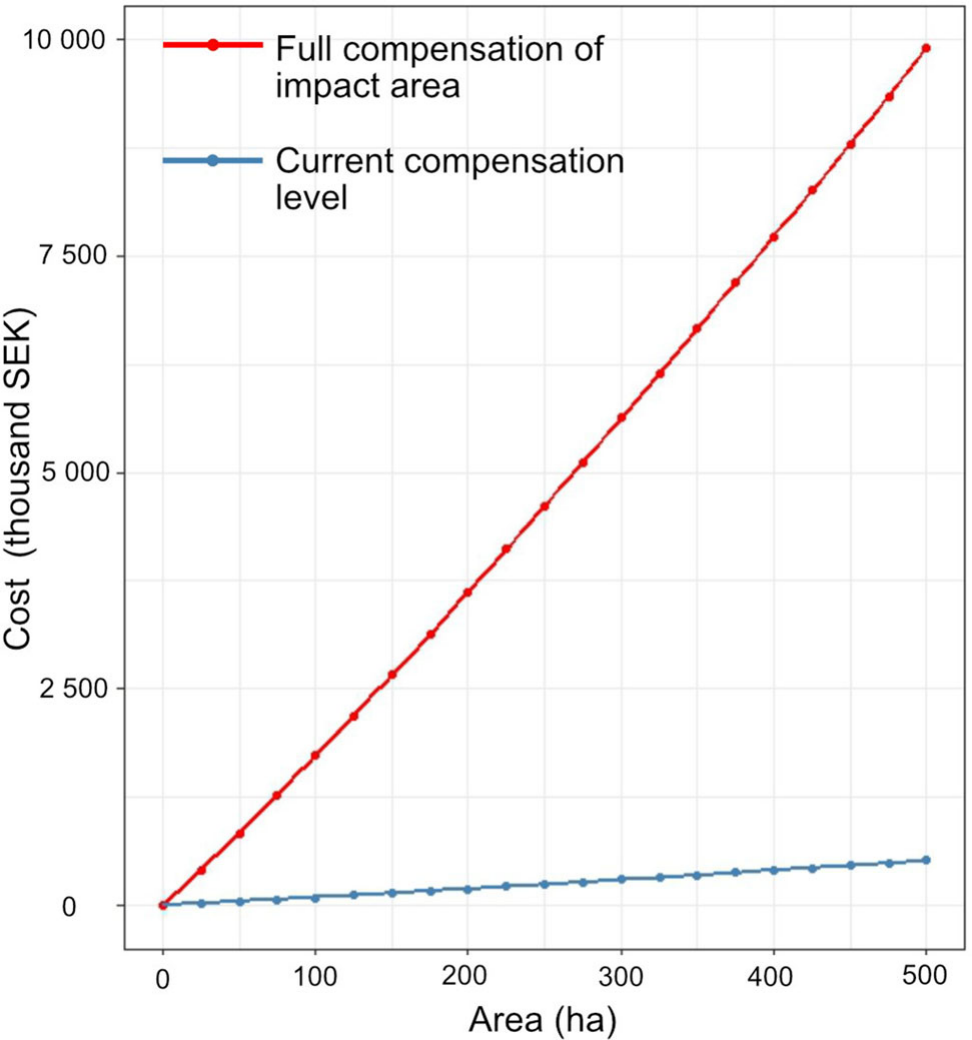
Relationship between size of area to compensate and the total costs of translocation, derived from functions in Lindroos et al. (2021). Current compensation level (blue line) of MDP and HDP pooled (equivalent to addition of 2.1 logs ha^−1^ in entire compensation area) and theoretical compensation level needed to reach full compensation, or deadwood volumes similar to those found in impact area (red line, equivalent to addition of 40.4 logs ha^−1^ in entire compensation area)

The total cost of translocating deadwood to compensate for deadwood loss increases with the size of both impact and compensation areas, as visualized by the theoretical example in Fig. 4. To compensate deadwood loss in a landscape context to the levels found in the impact area would require an addition of approximately 12 500 logs at a total cost of 5.8 million SEK, compared to the actually performed compensation measure in which 640 logs were translocated at a cost of 0.3 million SEK. To fully compensate on landscape level, it would, hence, require a 20 times higher effort in both number of logs and costs.

### Deadwood composition

**Fig. 5:**
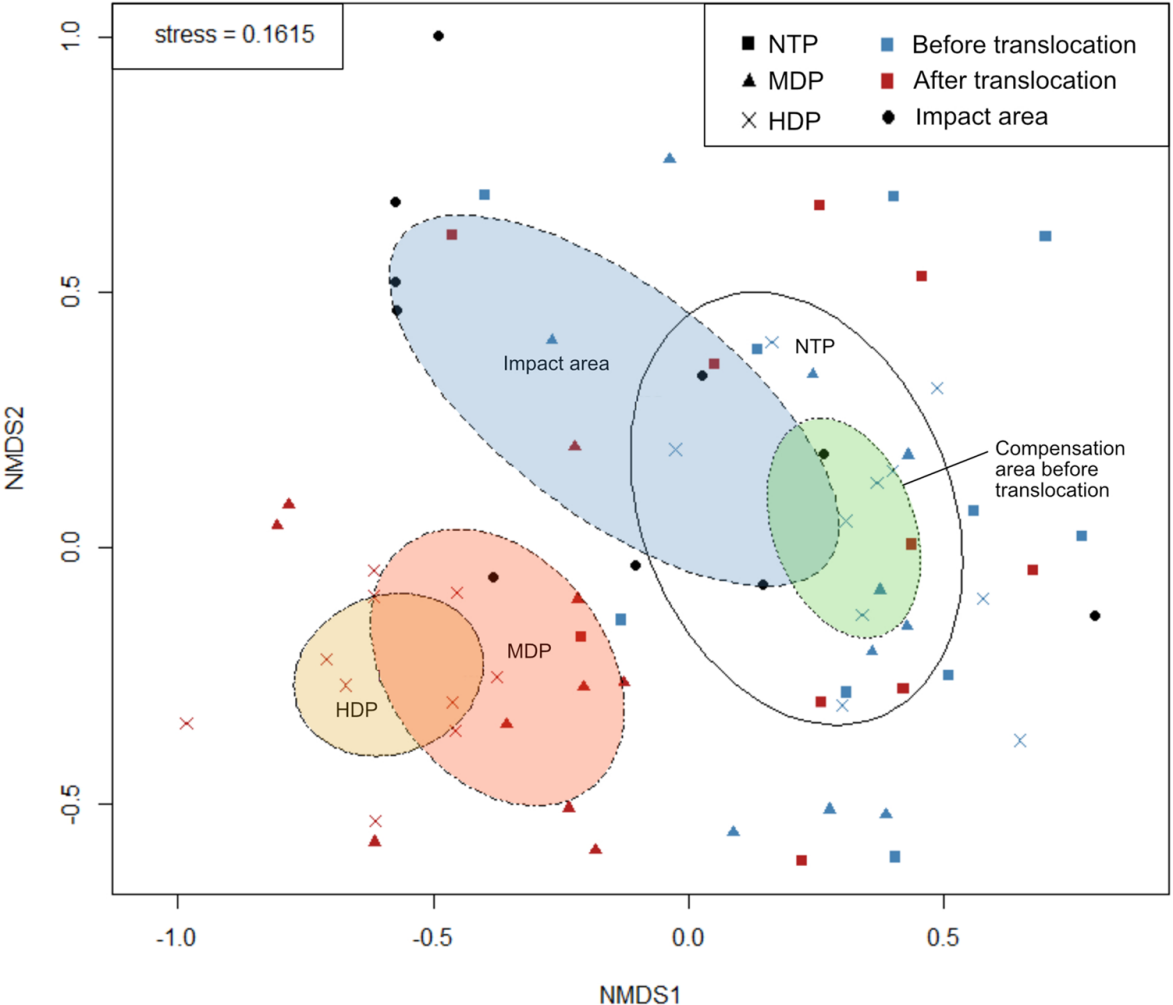
NMDS ordination results for the composition of deadwood substrates on plot level for the four studied treatment groups; impact area, no translocation plots (NTP), medium density plots (MDP) and high density plots (HDP). Color labels the temporal scale; blue symbols indicate plots before translocation, red symbols after translocation and black symbols the impact area that was cut down. Ellipses display the standard errors for the five groups

In total, 2086 individual deadwood substrates were identified across all examined plots, including both existing and translocated substrates. These were further categorized into 92 unique deadwood types out of a possible 240. Prior to translocation, the composition of unique deadwood types in the compensation area differed significantly from that in the impact area. Following translocation, we observed significant differences not only between the impact area and compensation treatment plots but also among the different translocation plots, as presented in Table 2 and Fig. 5.

### Indicator species analysis

Four specific deadwood substrate groups, pine snags in DC3 and with a diameter class of 20–30 cm (Kelo trees; decorticated and resin-impregnated long ago deceased pines), spruce snags in DC1 + DC2 with a diameter class of 30–40 cm, and downed willow in DC3 (hard) with a diameter class of 10–20 cm, were shown to be significant indicators of the impact area (Table 3). Willows were not translocated but they were frequent in the plots prior to translocation.

**Table 3:** Results of indicator species analysis for unique deadwood types consisting of tree species, decay class, diameter class and position. The table only includes substrate groups showing significant (*p* > 0.05) indicator value. Number of translocated substrates within parentheses marks the number of substrates belonging to a specific substrate group which was reclassified to another group after translocation, e.g., snags that after translocation were classified as downed deadwood

Area	Tree species	DC-class	Diameter class (cm)	Deadwood position	*p*-value	Number of substrates in compensation plots prior to translocation	Number of translocated substrates
Impact area	Pine	3	20–30	Standing	< 0.001	0	(12)
	Spruce	2	30–40	Standing	0.008	1	(18)
	Spruce	1	30–40	Standing	0.034	0	(98)
	Willow	3	10–20	Downed	0.006	1	0
Compensation area							
MDP	Pine	2	50+	Downed	0.032	0	4
HDP	Birch	3	10–20	Standing	0.032	109	0
	Pine	1	20–30	Downed	< 0.001	1	51
	Pine	1	30–40	Downed	< 0.001	0	77
	Pine	1	40–50	Downed	< 0.001	0	71
	Pine	1	50+	Downed	< 0.001	0	39
	Pine	2	20–30	Downed	< 0.001	0	21
	Pine	2	30–40	Downed	< 0.001	0	32
	Pine	3	40–50	Downed	0.018	3	11
	Spruce	1	20–30	Downed	< 0.001	4	108
	Spruce	1	30–40	Downed	< 0.001	2	98
	Spruce	1	40–50	Downed	< 0.001	0	41
	Spruce	1	50+	Downed	0.003	0	9
	Spruce	2	20–30	Downed	< 0.001	2	42
	Spruce	2	30–40	Downed	< 0.001	1	18
	Spruce	3	40–50	Downed	0.009	0	3

In the compensation area, MDPs had only downed pine in DC2 and with a diameter class of + 50 cm as a significant indicator, while the HDPs had the highest number of indicators. Among the 14 significant indicators, all except one were substrate groups that had been translocated. The remaining significant indicator was birch snags in DC3 with a diameter class of 10–20 cm, which had not been translocated but were frequent in the compensation plots prior to translocation.

**Fig. 6:**
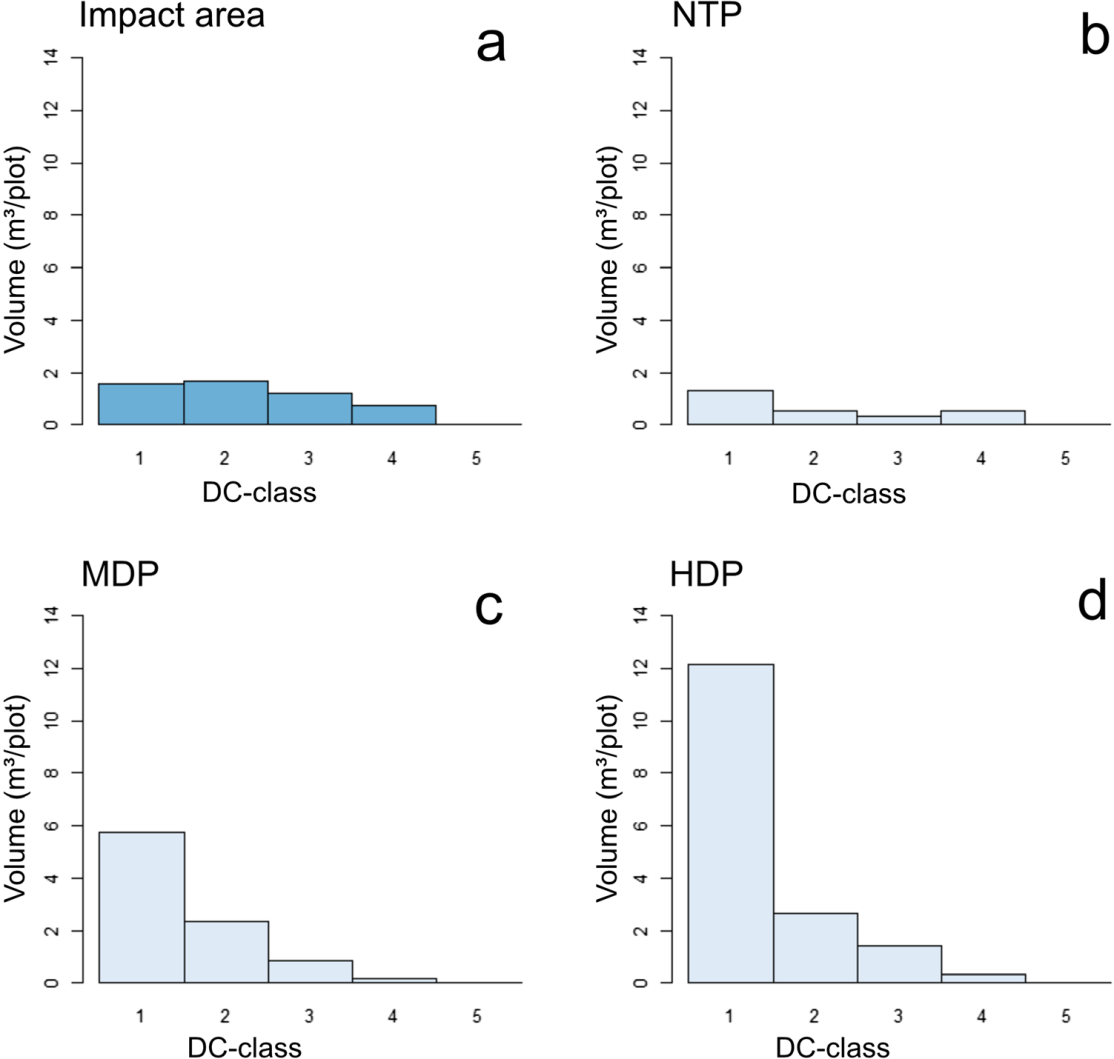
Decomposition class distribution before deadwood translocation in impact area (**a**) and NTP (**b**) and after translocation in MDP (**c**) and HDP (**d**)

The decomposition class distribution changed due to translocation. The relative volume of early decomposition class (classes 1 and 2) deadwood increased in the MDP and HDP (Fig. 6).

## Discussion

We assessed if this novel method for ecological compensation, translocation of high-quality deadwood, potentially could help solve long delivery time of certain substrates and structures, in our case large-diameter deadwood of unusual qualities. Translocation of deadwood and associated organisms is a method that has rarely been used and that has, to our knowledge, never been scientifically evaluated. This despite the fact that it is a potentially very useful method for advancing ecological compensation and restoration.

### Deadwood density at plot and stand scale

We found that translocation clearly increased the deadwood volumes in our experimental plots to levels well above (two to four times) compared to the impact area and equivalent to volumes found in deadwood rich old growth forest (Siitonen 2001). Furthermore, when we extrapolated the deadwood volumes observed in our experimental plots to the stand level, we found that the volumes in MDP and HDP were comparable (mean of 15.4–24.3 m^3^ ha^−1^) to those observed in the impact area (mean of 21.1 m^3^ ha^−1^). In comparison, Rudolphi and Gustafsson (2011), reported average deadwood volumes of 21 m^3^ ha^−1^ in old growth boreal forests that had not been subjected to clear-felling and contrasting to this, Jonsson et al. (2016) showed that the average deadwood volumes in production forests in northern Sweden is between 7.87 and 8.25 m^3^ ha^−1^, depending on region. Thus, purely based on volume, the method of deadwood translocation increased deadwood volumes in the MDP and HDP to levels equivalent or above levels found in targeted old growth forest. It is difficult to estimate volumes of deadwood needed to maintain intact communities of deadwood associated organism, however 20 m^3^ ha^−1^ is often used as rule of thumb for threshold volumes of deadwood, albeit rare and demanding saproxylic species might demand higher levels and specific qualities of deadwood (Müller and Bütler 2010). According to Hekkala et al. (2023), 20 m^3^ ha^−1^ is the threshold volume of deadwood for significantly higher richness of red-listed species in boreal coniferous forests. This indicates that purely based on volume, and not considering diversity, the levels of translocated deadwood would be sufficient to maintain population of rare and red-listed species in the compensation area at stand level.

The current translocation method with aggregation of high volumes of deadwood in experimental plots, could potentially work as “deadwood hotspots” in the landscape with high density and diversity of habitat for saproxylic species. Restoration, especially enrichment with deadwood, speeds up the development of the deadwood volumes needed to host large portions of biodiversity (Hekkala et al. 2016; Hägglund and Hjältén 2018) and potentially this method will circumvent the long delivery times for large dimension deadwood (Morris et al. 2006). These deadwood hotspots could possibly work as sources of dispersal in a metapopulational perspective (Ovaskainen and Hanski 2004) enriching the surrounding landscape. In ecological restoration, local enrichment with deadwood, attracts a large number of saproxylic insects and a high diversity of deadwood substrates that translates to a high diversity of saproxylic species (Hjältén et al. 2012; Lee et al. 2014; Hekkala et al. 2016; Seibold et al. 2017; Hägglund and Hjältén 2018).

One of the primary benefits of translocating high-quality deadwood is the assisted migration of rare species associated to these substrates. Restoration efforts like in situ deadwood creation require long timeframes to reach similar advanced decomposition stages and deadwood dependent communities might take long time to colonize through natural processes (Morris et al. 2006). Assisted migration of species, that would otherwise have been destroyed in impact areas, offers the possibility of preserving them and potentially establishing populations in new areas. Yet, the challenges persist: can they endure the translocation and thrive in new habitats and potentially colonize from translocated to local substrates? Though not explored here, this potential advantage demands further research and consideration.

An additional advantage is that translocation increases the amount of deadwood in the compensation area without reducing the standing tree volume of living trees. This enables trees within the compensation area to mature over time, subsequently contributing with higher amount of quality deadwood in the future.

### Costs for full landscape scale compensation and cost efficiency

Our calculations of the translocation effort needed to compensate for deadwood loss at larger spatial scales or landscape level, and the costs associated with this, revealed that a greater translocation effort is needed to fully compensate the losses of deadwood. In this respect, it should be noted that it was never the intention with this experiment to fully compensate for deadwood loss at landscape level, since this experiment was only a small part of the total compensation effort. Even so, our results show that it is possible to compensate for deadwood loss at smaller scales without extensive cost. Increasing the spatial scale also increases the difference between deadwood volumes added by the executed translocation level and deadwood volumes in the impact area. To fully compensate on landscape level, it would require a 20 times higher effort in both number of logs and costs compared to the executed compensation. Under the conditions in our theoretical example, the average additional cost for full compensation would be in the range of 16 000–20 000 SEK ha^−1^ for landscapes between 25 and 500 ha. Hence, the cost of 0.3 million SEK for the executed translocation would suffice for fully compensating an area of approximately 20 ha. To fully compensating the whole impact area of 376 ha would cost around 6 million SEK. This is of course a considerable cost, but should be seen in the perspective of the generally very high cost of these types of industrial projects. In this perspective the additional cost of increase the executed translocation level to fully compensate the habitat loss could not be considered remarkably high.

Furthermore, deadwood translocation costs can be considerably reduced by making the translocation process more efficient. The largest part of the executed compensation cost originated from the insertion of logs into the compensation area (29% of the cost), followed by log marking (24%) (Lindroos et al. 2021). If these types of work could be done more efficiently, it would substantially reduce the costs. That work is directly dependent on the volumes required to translocate, which in turn is dependent on the difference in deadwood densities between impact and compensation area. If the difference is little, deadwood from only a part of the impact area might be required. If so, deadwood close to the road could be collected, which would decrease costs. In addition to high amount of deadwood in the compensation area, it could also be selected based on its closeness to roads to decrease insertion distances and thereby costs. Since the cost for road transport was quite small per distance (5% of total cost in the executed translocation) (Lindroos et al. 2021), it would be cost-efficient to carefully select the compensation area without focusing too much on the closeness to the impact area. Additional measures would be to use a larger forwarder during insertion, and to place a larger proportion of the logs closer to the road. Aggregating deadwood into plots that are adapted to the loading capacity of the forwarder may also be more practical and cost-efficient in terms of creating higher deadwood density and diversity on a smaller number of plots.

However, as discussed in Lindroos et al. (2021), there is a trade-off between cost-efficiency and the created ecological values. Ongoing monitoring and research will reveal associated biodiversity benefits from aggregating deadwood into MDP versus HDP, revealing short- and long-term cost-efficiency of the methods. For large-scale exploitation projects, and as a last step in the mitigation hierarchy, substrate translocation can be a cost-efficient method provided that the loss of biodiversity is compensated.

### Deadwood composition

Deadwood diversity is strongly connected to diversity of wood-living organism (Hjältén et al. 2012; Lee et al. 2014; Seibold et al. 2017; Hägglund and Hjältén 2018). Maintaining a high diversity of deadwood is therefore instrumental for biodiversity conservation (Ulyshen and Hanula 2009; Toivanen and Kotiaho 2010; Stokland et al. 2012). Although the translocation of deadwood resulted in increased volumes of deadwood, we found that the composition of deadwood differed between the impact area and the experimental plots also after translocation. A higher number of translocated deadwood in the HDP did not reduce the dissimilarity in deadwood composition between the impact area and compensation plots, if anything it increased the differences in deadwood composition, depending on the selection of translocation substrates. Thus, translocation of deadwood will not automatically result in a similar deadwood composition as in an impact area.

Indicator species analysis revealed that three unique deadwood types had significantly higher density in the impact area; standing kelo trees of pine in DC3, standing recently dead spruce trees (DC1 and DC2), and down deadwood of willow in DC3 (slightly softened). Several factors contribute to this difference in deadwood composition between impact and compensation area. The clearest reason is the methodology used to translocate standing dead trees. When placed in the compensation plots the posture of standing deadwood was changed, since all translocated snags were downed. This could potentially lead to insufficient amount of standing deadwood and could have negative effects for saproxylic species dependent on this type of habitat. Deadwood stature has been shown to have a strong impact on the composition of deadwood living organism such as insect, wood fungi, bryophytes and lichens (Ulyshen and Hanula 2009; Toivanen and Kotiaho 2010; Hjältén et al. 2012; Stokland et al. 2012; Santaniello et al. 2017; Hägglund and Hjältén 2018). The conversion of standing kelo trees to downed logs make them unsuitable for species associated with standing deadwood (Santaniello et al. 2017). In future ecological compensation, or restoration projects involving translocation of deadwood, should be designed to maintain standing deadwood in vertical position even after translocation, alternatively combined with in situ creation of standing dead trees. Problems related with this include increased costs, practical and safety considerations since placing and maintaining large deadwood in a standing position in a safe way is not an easy task. However, methods to move standing deadwood and keep it standing with support from living trees on sites have been tried in smaller scales so evaluation of these trials together with method development could make this possible in the future.

In the case of deadwood from willows, this type of deadwood was not translocated (only pine and spruce) and willows were rare in the compensation area, which explains the higher density in the impact area. As deciduous trees harbors saproxylic communities distinct from conifers (Jonsell et al. 2007; Müller et al. 2015; Kärvemo et al. 2023) the lack of deadwood from willows in the compensation area could result in a lower species richness and abundance of willow-associated species. Thus, no-net-loss for this species group will not be achieved and further detailed studies are needed to assess translocation of deciduous trees. This could be an important future conservation measure, since other studies have revealed a strong positive correlation between availability of deadwood from deciduous trees and associated saproxylic species (Johansson et al. 2017).

The positive aspect of translocation was that many substrate types became more common in the compensation area than in the impact area. The indicator species analyses revealed that many of the deadwood indicators of HDPs belonged to DC1–2. This is a direct result of the fact that the selection of suitable deadwood substrates for translocation was limited to substrates that would not break during transport. In fact, many of the translocated substrates in early decomposition classes origins from cut living trees of high conservation values (very old and with large diameter). This resulted in an overrepresentation of deadwood in early decomposition stages, omitting more decomposed substrates. This means large amounts of habitats have been translocated for early successional saproxylic species. With time, these substrates in early decomposition classes will progress to more advanced decay classes and thereby serve as habitat for late successional species, even those demanding large diameter deadwood (Juutilainen et al. 2011). The transformation of living trees with high conservation value to early decay deadwood means that the rich abundance of old trees was not compensated for. One way to compensate for lack old trees, diversity in tree microhabitats (e.g., scars, resin flows, hollows), is to apply veteranisation on trees in the compensation area, e.g. by bark stripping.

Translocation increased the occurrence of a type of deadwood substrates that are becoming extremely rare in the forest landscape, large diameter deadwood (> 30 cm in diameter) (Fridman and Walheim 2000) but that are regarded as beneficial for many specialist saproxylic species (Juutilainen et al. 2011). Thus, in this case translocation not only increased deadwood volume in the compensation area but potentially also quality of deadwood compared to the surrounding forest matrix.

The indicator species analyses also revealed that deadwood of birch and large diameter downed logs were indicators of HDPs. As no birch was translocated this result is explained by a higher occurrence of birch deadwood in the compensation area than in the impact area. As deadwood of birch harbor a large variety of saproxylic organisms and a different species community than conifers (Stokland et al. 2012; Bell et al. 2015; Hägglund and Hjältén 2018), this also potentially contributes to the overall biodiversity in the compensation area.

## Conclusions and practical implications

Translocation of deadwood in ecological compensation areas could be viewed as a new tool for forest restoration and ecological compensation, reducing long delivery times of high-quality substrates, as well as providing means to improve colonization of deadwood associated species to a new area. Our results show that using this method, deadwood volumes at stand level would reach levels around or above the 20 m^3^ ha^−1^, suggested as a threshold for maintaining high species richness of rare and threatened saproxylic species. However, as translocation of deadwood is a novel method rarely used and very poorly evaluated, it requires improvements. Based on experiences from this case study we stress the importance of increasing the selection of suitable translocation substrates, but also to evaluate compensation strategies that combines translocation with in situ creation or veteranization of deadwood, to include the full range of deadwood decomposition classes and tree species, as far as possible. New translocating methods for late decay downed deadwood and standing deadwood need to be developed and evaluated. Further, it is important to try to maintain standing deadwood standing also after translocation, so this type of substrate does not become unsuitable (i.e., an ecological trap in connection with translocation) for associated organisms following translocation.

Full compensation of deadwood volumes on scales similar to the impact area is resource demanding but our calculations show that even without methodological improvements the costs are low in comparisons to the budgets of large-scale exploitation projects such as a mine expansion. Provided that the desired biodiversity benefits are achieved, such compensation methods can be considered efficient. However, provided the novelty of the methods, cost-related improvements could most likely be made. Hence, there is a need to investigate how the cost-efficiency could be increased, while maintaining the desired biodiversity benefits.

To gain further essential knowledge of this novel compensation method it is of outmost importance to conduct monitoring of the fate of the species communities moved with the translocated high-quality logs, e.g., their ability to colonize available substrates in the compensation area. Such monitoring programs should be given high priority as they are essential for assessing the potential biodiversity benefits with translocation. Finally, our findings emphasizes a broader principle of ecological compensation, applicable to all comparable impacts, such as urban and infrastructure development. The optimal strategy should be to preserve the most valuable habitats from irreversible harm. Only when preservation is impossible due to societal justifications for the impact outweigh conservation concerns should compensatory actions, including translocation, be pursued.
