# Correction to: Translocation of deadwood in ecological compensation: A novel way to compensate for habitat loss

**DOI:** 10.1007/s13280-023-01973-7

**Published:** 2024-01-13

**Authors:** Olov Tranberg, Anne-Maarit Hekkala, Ola Lindroos, Therese Löfroth, Mari Jönsson, Jörgen Sjögren, Joakim Hjältén

**Affiliations:** 1 Department of Wildlife, Fish, and Environmental Studies, Swedish University of Agricultural Sciences, 901 83 Umeå, Sweden; 2 Department of Forest Biomaterials and Technology, Swedish University of Agricultural Sciences, 901 83 Umeå, Sweden; 3 SLU Swedish Species Information Centre, Swedish University of Agricultural Sciences, Uppsala, Sweden

**Correction to: Ambio** 10.1007/s13280-023-01934-0

In the original publication of the article, one of the equations presented in the text under the section “Data processing and analysis” was missing a vital part. It concerns the presentation on how to calculate the well-known geometrical form of a truncated cone.

Below are the correct and the uncorrected version of the equation.

***As presented (wrong) in the article:***

$${V}_{\text{t}}=\left(L* \frac{\pi }{3}\right)*\left({{r}_{\text{max}}}^{2}+{r}_{\text{min}}*{{r}_{\text{min}}}^{2}\right).$$

***Correct version as it should be presented:***

$$V_{\text{t}}=\left(L* \frac{\pi }{3}\right)*({{r}_{\text{max}}}^{2}+{r}_{\text{max}}*{r}_{\text{min}}+ {{r}_{\text{min}}}^{2})$$

The original article has been corrected.

